# Evaluating whole genome sequence data from the Genetic Absence Epilepsy Rat from Strasbourg and its related non-epileptic strain

**DOI:** 10.1371/journal.pone.0179924

**Published:** 2017-07-14

**Authors:** Pablo M. Casillas-Espinosa, Kim L. Powell, Mingfu Zhu, C. Ryan Campbell, Jessica M. Maia, Zhong Ren, Nigel C. Jones, Terence J. O’Brien, Slavé Petrovski

**Affiliations:** 1 Department of Medicine, The Royal Melbourne Hospital, The University of Melbourne, Parkville, Victoria, Australia; 2 Duke University School of Medicine, Duke University, Durham, North Carolina, United States of America; 3 BD Technologies, Research Triangle Park, Durham, North Carolina, United States of America; 4 Institute for Genomic Medicine, Columbia University Medical Center, Columbia University, New York, New York, United States of America; University of Modena and Reggio Emilia, ITALY

## Abstract

**Objective:**

The Genetic Absence Epilepsy Rats from Strasbourg (GAERS) are an inbreed Wistar rat strain widely used as a model of genetic generalised epilepsy with absence seizures. As in humans, the genetic architecture that results in genetic generalized epilepsy in GAERS is poorly understood. Here we present the strain-specific variants found among the epileptic GAERS and their related Non-Epileptic Control (NEC) strain. The GAERS and NEC represent a powerful opportunity to identify neurobiological factors that are associated with the genetic generalised epilepsy phenotype.

**Methods:**

We performed whole genome sequencing on adult epileptic GAERS and adult NEC rats, a strain derived from the same original Wistar colony. We also generated whole genome sequencing on four double-crossed (GAERS with NEC) F_2_ selected for high-seizing (n = 2) and non-seizing (n = 2) phenotypes.

**Results:**

Specific to the GAERS genome, we identified 1.12 million single nucleotide variants, 296.5K short insertion-deletions, and 354 putative copy number variants that result in complete or partial loss/duplication of 41 genes. Of the GAERS-specific variants that met high quality criteria, 25 are annotated as stop codon gain/loss, 56 as putative essential splice sites, and 56 indels are predicted to result in a frameshift. Subsequent screening against the two F_2_ progeny sequenced for having the highest and two F_2_ progeny for having the lowest seizure burden identified only the selected *Cacna1h* GAERS-private protein-coding variant as exclusively co-segregating with the two high-seizing F_2_ rats.

**Significance:**

This study highlights an approach for using whole genome sequencing to narrow down to a manageable candidate list of genetic variants in a complex genetic epilepsy animal model, and suggests utility of this sequencing design to investigate other spontaneously occurring animal models of human disease.

## Introduction

The genetic generalised epilepsies (GGE) comprise a group of epileptic disorders that include absence seizures as one of the classical seizure phenotypes [1–3] with evidence that some cases might be explained by a complex polygenic mode of inheritance [4–10]. Absence seizures are characterized by diffuse cortical bilateral 3Hz spike-and-wave discharge (SWD) on electroencephalogram (EEG) [1]. The Genetic Absence Epilepsy Rats from Strasbourg (GAERS) are a well-validated rat model of spontaneous GGE with absence seizures that were selectively bred from a Wistar colony that displayed spontaneous absence seizures [11, 12]. In parallel, a counter strain was also bred from the same original colony, the Non-Epileptic Control (NEC) rats, which were selectively bred for the lack of seizures and SWDs [12, 13]. Analogous to human absence epilepsy, GAERS have spontaneously occurring SWDs that start and end abruptly on a normal EEG background [5, 12, 13]. GAERS also exhibit a similar pharmacological profile when compared to human absence seizures, being suppressed or aggravated by the same anti-epileptic drugs [12–18]. Moreover, GAERS exhibit depressive-like behaviour and elevated anxiety, analogous to the human behavioural profile of GGE [19–21]. Extensive cross breeding studies have demonstrated that the GAERS epileptic phenotype is likely to be complex and polygenic [12]. A genome-wide linkage scan of GAERS x Brown Norway (BN) F_2_ rats reported three quantitative trait loci (QTL) in chromosomes 4, 7, and 8, associating with specific aspects of the SWD phenotype [22]. Despite three decades of study, only one GGE-associated genetic variant has been identified that has been implicated with the epilepsy phenotype. We have published the missense (R1584P) variant in the low-threshold T-type calcium channel Ca_V_3.2 gene (*Cacna1h*) as accounting for up to 33% of variance for the percentage of time in seizures and the number of seizures based on data from F_2_ progeny of a cross between NEC and GAERS rats [23, 24].

Taken together, the GAERS and NEC rats represent a powerful opportunity to identify neurobiological factors associated with the GGE phenotype. Here, we performed whole genome sequencing (WGS) on these two strains that have been selectively bred from the original colony to characterise the genomic differences between them. We present the sequence variation identified among the GAERS, NEC and their F_2_ progeny selected for high and non-seizing phenotypes, using short-read next-generation sequence technology. We report a near complete catalogue of strain-specific variants between the affected and unaffected strain, and each compared to the reference BN genome.

## Materials and methods

### Ethics statement

All procedures on rats were approved by The University of Melbourne Animal Ethics committee (ethics numbers 1011823) and followed the Australian Code of Practice for the care and use of animals for scientific purposes.

### EEG electrode implantation surgery

To identify the SWD phenotype, rats were anesthetised with isoflurane, then single midline incision was made on the scalp posterior to the eyes to between the ears. Six holes were drilled through the skull without penetrating the dura, one on each side anterior to the bregma, two on each side posterior to the bregma and two to each side anterior to lambda. Recording electrodes were screwed into each hole. Each recording electrode comprised a 1.3 mm gold connector (Farnell Components, Chester Hill, Australia) soldered onto a nickel alloy jeweller screw. The recording electrodes were fixed in position by applying Vertex dental cement around the electrodes and over the skull. The incision was then sutured using nylon (4/0). Immediately after surgery, each rat received an intraperitoneal injection of 1 ml/kg analgesic solution containing intraperitoneal carprofen analgesic (5 mg/kg; Rimadyl; Pfizer Australia) in 0.9% sodium chloride. Post-surgical animals were assessed for neurological, weight and overall appearance changes twice daily for the first 5 days following surgery, and then every other day thereafter. Carprofen analgesia was given twice a day during the first 3 days after the surgery. Animals are humanely euthanized if they present sustained signs of suffering during the course of the experiments. All the animals used in our experiments had a satisfactory and prompt recovery after the surgery.

### Animals

We whole-genome sequenced female GAERS (n = 2), NEC (n = 2) and F_2_ GAERS x NEC (n = 4) rats aged 16 weeks from our breeding colonies in Royal Melbourne Hospital. The rats were originally obtained from the Strasbourg colony in 2007 (i.e. GAERS STRAS-MELB [24]). The GAERS and NEC lines used for sequencing are at F_82_ and F_74_ filial generations, respectively. All procedures were approved by the University of Melbourne Animal Ethics Committee (#1011823).

### Breeding of the F_2_

The double cross matings used in this study were bred based on the Ca_V_3.2 R1584P variant. [23] First, GAERS homozygous for the R1584P variant were crossed with NEC rats that were homozygous for the major allele to produce an F_1_ generation. Then, two F_1_ generation rats were mated to produce an F_2_ generation. On average, 25% of the F_2_ progeny would be expected to be homozygous for a given GAERS or NEC variant, 50% heterozygous for the variant, and 25% to not carry the variant [23].

### EEG recordings and spike and wave discharge analysis

Two weeks after surgery, the animals underwent two 24-hours periods of continuous EEG recording with at least 2 days between each recording to confirm the epileptic phenotype. EEG recordings were obtained using Profusion 3 software (Compumedics, Australia) unfiltered and digitized at 512Hz.

EEG recordings were analysed for SWDs to give an electroencephalographic confirmation of absence seizures. The analysis of the EEG was blinded using the SpikeWave Complex Finder software (SWC, PLC van den Broek, Nijmegen University, Netherlands). The seizures detected were confirmed manually using the following criteria: a SWD complex with an amplitude 3 times greater than the baseline, SWD frequency of 7–12 Hz and SWD duration longer than 0.5s, as previously described [24]. The seizures detected were confirmed manually using the GAERS’ standard criteria for the classification of seizures, which includes; total number of seizures, total time spent in seizure activity and average seizure duration were analysed. No seizures (or spike-wave discharges) were detected among our NEC rats (evaluated up until 12 months of age) or the no-seizing F_2_ rats.

### Tissue collection

2–5 days after the last EEG recording session, animals were anesthetized using isoflurane at 5% (Ceva isofluorane, Piramal Enterprises Limited, India), then culled using a lethal dose of intraperitoneal injection of lethabarb (150mg/kg IP; pentobarbitone sodium; Virbac, Aus.). Liver and brain tissue where collected, snap frozen over liquid nitrogen and stored at -80°C.

### Extraction of genomic DNA

Genomic DNA (gDNA) was extracted from liver using the DNeasy Blood & Tissue Kit (QIAGEN) following the manufacturer’s protocol. Samples were incubated for 2 hours at 37°C with proteinase K (0.4μg) to digest the tissue and treated with 200μg of RNase to degrade any contaminating RNA. Once extracted, gDNA was stored at -80°C.

### Genome sequencing

We sequenced the GAERS and NEC genomes using paired-end sequencing libraries on an Illumina HiSeq 2000 machine. We used the rat reference genome, version 3.4 (*rattus norvegicus*), which represents >90% of the rat genome. Four female F_2_ animals were selected for whole genome sequencing based on the number of seizures that they displayed. Two of the F_2_ animals had no seizures and did not carry the Ca_V_3.2 R1584P variant (non-seizing F_2_). The other two F_2_ animals selected were homozygous for Ca_V_3.2 R1584P variant and displayed the highest number of seizures per hour, 5.8 and 4.6 seizures respectively (high-seizing F_2_).

### Concordance with genotyping chips

Rats from the GAERS and NEC strains have been independently genotyped in 2008 by the STAR consortium using a single-nucleotide polymorphism (SNP) rat genotyping microarray [25]. Of the 20,283 STAR consortium SNPs [25], 11,379 and 10,830 SNPs had been determined to be polymorphic between the BN reference and the GAERS and NEC strains, respectively.

At QUAL PHRED consensus score ≥30 and read depth ≥3, the sequencing data from the GAERS and NEC detected 11,292 and 10,648 of the 11,379 and 10,830 SNPs previously assigned as polymorphic (99.24% and 98.32% sensitivity). Of the 8,475 and 8,624 positions previously assigned as non-polymorphic, our sequencing data correctly assigned 8,408 and 8,440 as non-polymorphic (99.21% and 98.29% specificity). These results reflect high concordance rates between the previous genotyping array and our own Illumina HiSeq sequencing data. The minor discordances can be explained by differences in technologies and variant calling platforms resulting in discordant variant calls at the false positive and false negative variant sites. That the animals sequenced are being compared to genotype data from rats maintained in a different laboratory for the last 20 generations would also result in minor discordance. For the 67 (GAERS) and 184 (NEC) apparent false-positive SNPs, the sequencing consensus quality (QUAL) score was high (mean score 58.22 and 58.30) with close-to-average read depth (average depth 13.70 and 15.44 reads). Interestingly, for 43 (64.2% GAERS) and 43 (23.4% NEC) of the false positive (FP); and, 42 (48.28% GAERS) and 43 (23.63% NEC) of the apparent false negatives (FN), the STAR BN/SsNHsd/Mcwi allele were also discordant with the BN reference genome, suggesting that this subset of apparent FP and FN SNPs may actually reflect residual sequencing errors in the BN reference genome (RGSC-3.4).

## Results

### Data access

The data sets supporting the results of this research article are included within the article; the supporting additional files are presented as supplementary tables (S1–S6 Tables).

### Sequencing

Whole genome sequencing of rat DNA was performed on the Illumina Hiseq 2000. To determine coverage, all gaps (stretches of N's) in the rat reference genome (NCBI *rattus norvegicus* RGSC v3.4 (b4); Ensembl core database release 67) were excluded, resulting in a reference of 2,718,881,021 bases. After accounting for PCR duplicates and reads that did not align to the reference genome, in the GAERS, approximately 23.48 million reads (37.6 Gb) were mapped to the BN reference genome (RGSC v3.4) using BWA [26] and variant calling using samtools [27]. This resulted in 15.14-fold average coverage of the GAERS autosomal chromosomes, and 15.65-fold average coverage of the X-chromosome. Similarly, for the NEC rat, 26.48 million reads (42.7 Gb) mapped to the BN reference genome, equating to 17.2-fold average coverage of the NEC autosomal chromosomes, and 17.57-fold average coverage of the X-chromosome (Table 1). In total, within the GAERS sequence, 98.2%, 96.9%, and 84.2% of non-gap, non-N bases of the autosomal reference genome were covered by at least three, five or ten reads, respectively. Similarly, for the NEC sequence, 98.4%, 97.5%, and 90.3% of non-gap, non-N bases of the autosomal reference genome were covered by more than three, five, or ten reads, respectively (Table 1).

**Table 1:** Coverage details.

Region	Strain (n = 2 each)	Average read depth	%bases >1 read	%bases >3 reads	%bases >5 reads	%bases >10 reads	%bases >15 reads	%bases >20 reads
**Autosomal**	GAERS	15.14	99.1%	98.2%	96.9%	84.2%	48.3%	15.7%
	NEC	17.20	99.1%	98.4%	97.5%	90.3%	63.9%	27.6%
**X chromosome**	GAERS	15.65	99.1%	98.1%	96.7%	86.3%	54.8%	20.4%
	NEC	17.57	99.2%	98.4%	97.4%	90.9%	68.0%	32.5%

The homozygous variant calls specific to each strain were: 1,119,180 and 1,051,188 single nucleotide variants (representing 0.04% of bases covered with ≥3-fold coverage and a quality score ≥30), in GAERS and NEC, respectively. There were 296,455 (GAERS) and 298,996 (NEC) strain-specific putative insertions-deletions (indels) (Table 2).

**Table 2:** Variant tallies compared to BN rat reference (RGSC v3.4 b4).

Strain (n = 2 each)	Variant type	Variants	Homozygous Variants	Strain-specific variants* (%)
**GAERS**	SNV	3,910,940	3,713,694	1,119,180 (30.14%)
	Indel^#^	1,000,916	954,008	296,455 (31.07%)
**NEC**	SNV	3,863,956	3,663,472	1,051,188 (28.69%)
	Indel^#^	1,022,912	967,666	298,996 (30.90%)

Contributing variants were required to have ≥3-fold coverage and a PHRED QUAL (quality consensus) score ≥30.

*Homozygous variants specific to corresponding strain, after excluding variants present in the other strain. Variants where the other strain had insufficient (<3-fold) coverage or (QUAL<30) quality were omitted.

^#^Indels that overlapped a gap region in the rat reference genome were excluded.

### Distribution of variants

Among the called single nucleotide variants (SNVs): 3,571 of 1,119,180 (0.32%) and 3,224 of 1,051,188 (0.31%) were GAERS and NEC specific non-synonymous variants with annotations of missense, nonsense, or donor/acceptor splice variants. While 307 of 296,455 (0.10%) and 394 of 298,996 (0.13%) putative indels were GAERS and NEC specific variants with annotations of missense, nonsense, donor/acceptor splice, complex indel or frameshift. Both SNVs and indels were annotated using Ensembl variant effect predictor v2.5, based on Ensembl release v67, *rattus norvegicus*. Distribution of the annotations from the GAERS and NEC specific variants are available in (Tables 3 and 4).

**Table 3:** Annotations for single nucleotide variants observed only in one strain.

Annotation (SNVs)	GAERS—Specific (%)	NEC—Specific (%)
**All variants**	1,119,180	1,051,188
**Stop gain**	34 (0.0030%)	40 (0.0038%)
**Stop loss**	5 (0.00045%)	3 (0.00029%)
**Splice donor/acceptor site**	53 (0.0047%)	47 (0.0045%)
**Missense**	3,479 (0.31%)	3,134 (0.30%)
**Coding unknown**	12 (0.0011%)	16 (0.0015%)
**Within noncoding gene**	836 (0.075%)	848 (0.081%)
**Mature miRNA**	7 (0.00063%)	5 (0.00048%)
**Synonymous**	4,695 (0.42%)	4,300 (0.41%)
**Splice region variant**	604 (0.054%)	582 (0.055%)
**5’ UTR**	590 (0.053%)	464 (0.044%)
**3’ UTR**	2,855 (0.26%)	2,539 (0.24%)
**Upstream**	55,018 (4.92%)	57,203 (5.44%)
**Downstream**	54,056 (4.83%)	50,245 (4.78%)
**Intronic**	273,631 (24.45%)	252,563 (24.03%)
**Intergenic**	723,305 (64.63%)	679,199 (64.61%)

While a single variant can have multiple annotations due to overlapping transcripts, the variant annotations are presented such that a single variant only contributes to a single annotation, based on the order of the presented annotations.

**Table 4:** Annotations for indel variants observed in one strain and not the other, excluding indels overlapping non-reference sequence.

Annotation (Indels)	GAERS—Specific (%)	NEC―Specific (%)
**All variants**	296,455	298,996
**Stop gain**	1 (0.0003%)	0 (0.00%)
**Splice donor/acceptor site**	64 (0.022%)	74 (0.025%)
**Frameshift**	149 (0.05%)	204 (0.07%)
**Codon Indels**	121 (0.04%)	151 (0.05%)
**Within noncoding gene**	99 (0.033%)	127 (0.042%)
**Mature miRNA**	11 (0.0037%)	19 (0.0064%)
**Splice region variant**	202 (0.068%)	244 (0.082%)
**5’ UTR**	92 (0.030%)	85 (0.028%)
**3’ UTR**	836 (0.28%)	793 (0.27%)
**Upstream**	18,009 (6.07%)	18,240 (6.10%)
**Downstream**	14,896 (5.02%)	15,430 (5.16%)
**Intronic**	79,180 (26.71%)	80,114 (26.79%)
**Intergenic**	182,795 (61.66%)	183,515 (61.38%)

While a single variant can have multiple annotations due to overlapping transcripts, the variant annotations are presented such that a single variant only contributes to a single annotation, based on the order of the presented annotations.

### Strain-specific variants

To enrich for higher confidence protein-coding calls we took the 3,571 GAERS-specific and 3,224 NEC-specific non-synonymous SNVs and focused on those variants where the sequence data of the corresponding strain achieved all of the following: a) ≥10-fold coverage, b) a QUAL score ≥ 30, c) a mapping quality (MQ) score ≥ 40, d) a genotype quality (GQ) score ≥ 20 and e) ≥ 80% of total reads supported the variant allele (See S1 Table for GAERS and S2 Table for NEC). We further screened these higher confidence GAERS (n = 2,270) and NEC (n = 2,285) specific non-synonymous SNVs across other known rat variant databases: STAR rat consortium, dbSNP, Ensembl, the SHR rat sequence, and two more recently published consortia sequencing studies [28–32]. Herein, we refer to variants found in GAERS but not NEC, and vice versa, as “specific” variants. Variants that are also absent among the available rat variation reference cohorts are referred to as GAERS or NEC “private” variants. When screening out variants previously reported within external rat strains, the number of GAERS and NEC private SNVs with non-synonymous annotations was 183 and 168 SNVs, respectively (Table 5).

**Table 5:** Mapping rat non-synonymous SNVs to the human genome.

Criteria	GAERS-specific	NEC-specific
High Confidence non-synonymous variants*	2,270 (100%)	2,285 (100%)
**Successful in Liftover to GRCh37/hg19**	2,060 (90.75%)	2,096 (91.73%)
**Not observed in STAR SNP panel, dbSNP, Ensembl, or SHR rat sequence**	1,034 (45.55%)	1,128 (49.37%)
**Absent among rat strains sequenced as part of Atanur et al (2013) or Hermsen et al (2015)**.	183 (8.06%)	168 (7.35%)

* High confidence variants are defined by the sequence data of the corresponding strain achieving: a) ≥10-fold coverage, b) a QUAL score ≥ 30, c) a mapping quality (MQ) score ≥ 40, d) a genotype quality (GQ) score ≥ 20 and e) ≥ 80% of reads supporting the variant allele (See S1 Table for GAERS and S2 Table for NEC).

Of the strain specific high-confidence non-synonymous SNVs we found that 2,060 (90.75%) and 2,096 (91.73%) GAERS and NEC-specific SNVs successfully mapped from the rat (Nov. 2004 [Baylor 3.4/rn4]) to the human (Feb. 2009 [GRCh37/hg19]) genome using LiftOver [33].

Of the 307 and 394 GAERS- and NEC-specific protein-coding indels variant calls, 110 and 154 passed the stringent quality control criteria as previously described for the SNV calls. Of those, 78 GAERS- and 123 NEC-specific indels were located outside of repeat regions as defined by the *rattus norvegicus* (rn4) repeatmasker, accessed from the University of California, Santa Cruz (UCSC) Genome Browser (https://genome.ucsc.edu/). (See S3 Table for GAERS- and S4 Table for NEC-specific protein-coding indels).

### Copy number variants

We used the estimation by read depth with single-nucleotide variants (ERDS) platform, based on read depth and paired-end mapping data [34] to identify copy number variants (CNVs) among the GAERS and NEC sequence data. To qualify, CNV calls by ERDS were required to be ≥5000bps and to result in either complete loss of sequence (for deletions) or at least 2-fold increase in instance (for duplications). As a result, 354 and 475 putative GAERS- and NEC-specific CNVs were identified (S5 Table). Of the GAERS-specific putative CNVs, 39 (29 duplications and 10 deletions) are annotated to overlap with an exonic/splicing (coding) function. Similarly, 30 NEC-specific putative CNVs (22 duplications and 8 deletions) overlap with exonic/splicing coding sequence (S6 Table). CNVs of increasing size, as provided in field “length” in S6 Table, are of increased confidence, however, all CNVs reported in S5 and S6 Tables represent putative CNV calls and no CNVs have been independently validated by additional sequencing technologies.

No GAERS or NEC CNV was found to overlap with a list of genes characterized by a “seizure” associated phenotype among the Mouse Genome Informatics (MGI) and/or with the list of candidate epilepsy genes as defined by Lemke and colleagues [35].

### F_2_ variant screen

GAERS rats were crossed with NEC rats to produce an F_1_ generation. Heterozygous F_1_ generation rats were then mated to produce an F_2_ generation. To further identify variants with potentially large effects, we compared the presence or absence of the 183 GAERS private variants among four whole-genome sequenced F_2_ rats selected at the extremes of the GAERS and NEC seizure phenotype spectrum (i.e. two high-seizing and two non-seizing F_2_ rats respectively), and selected on the presence and absence of the previously reported *Cacna1h* variant. Of the GAERS private variants, no additional non-synonymous variant beyond the *Cacna1h* variant was homozygous among high-seizing F_2_ and absent among non-seizing F_2_ rats. We did not find any candidate variants that could be associated with epilepsy when we screened the 168 private NEC variants in which the non-seizing F_2_ were homozygous and absent among the high-seizing F_2_.

We then relaxed the requirement of carrier zygosity and co-occurrence with published rat strains to permit identifying risk variants that may be present among other strains but not be sufficient to cause the seizure phenotype alone. Of the 2,270 higher-confidence GAERS-specific SNVs, thirty-three variants were found exclusively among the two high-seizing F_2_ mice—irrespective of zygosity (S1 Table). For this zygosity permissive screen we then required that less than ~10% of the previously sequenced rat strains—none of which have reported to have a GAERS epileptic and behavioural phenotype—shared the variant (http://rgd.mcw.edu/pub/strain_specific_variants/). Thus, to qualify, the segregating GAERS-specific variant must also be observed in less than four of the 28 rat genomes published by Atanur et al [31] and also in less than five of the 40 strains recently published in Hermsen et al. [32]. Through this more permissive screen we identified six rare GAERS-specific non-synonymous SNVs that were transmitted to the two high-seizing but not the two non-seizing F_2_ animals, and were observed in less than 10% of the other published rat strains. Aside from the pre-selected *Cacna1h* variant, no variant occurred in a gene of obvious biological relevance. Using similar criteria we found fourteen NEC-specific non-synonymous SNVs transmitted to both non-seizing F_2_ but not the two high-seizing F_2_ animals (Table 6). We found that 13 of these 14 NEC-specific non-synonymous SNVs were located within a large block of 52.2Mbp on chromosome 7 from positions 69181224–121380101 (rn4 reference genome). The relevance of this stretch of sequence to the non-epileptic phenotype is of interest for larger F_2_ sample sequencing studies.

**Table 6:** 

Strain	Gene	Variant (rn4)	Hg19 (Lift Over)	Observed: Atanur 2013 (n = 28)	Observed: Hermsen 2015 Tally (n = 40)
GAERS	*Enc1*	chr2:27724615C>G	*ENC1*	n = 2	n = 4
GAERS	*Tmem82*	chr5:160516635G>A	*TMEM82*	n = 1	n = 2
GAERS	*Rsl1d1*	chr10:4315352C>T	*RSL1D1*	n = 2	n = 2
GAERS	*Rsl1d1*	chr10:4325924A>C	*SMOC1*	n = 2	n = 2
GAERS	*D3ZL18_RAT*	chr10:5565552G>A	*APOO*	n = 3	n = 4
GAERS	*Cacna1h*	chr10:14629759C>G	*CACNA1H*	None	None
NEC	*Ntrk3*	1:133959580A>G	*NTRK3*	None	None
NEC	*F1M6H9_RAT*	7:69181224A>G	*CTBS*	None	None
NEC	*F1M6H9_RAT*	7:69181798A>G	*SPATA1*	None	n = 1
NEC	*F1LXS3_RAT*	7:69870734C>A	*MATN2*	None	n = 1
NEC	*LOC681820*	7:69915154G>A	*C8orf47*	None	n = 1
NEC	*LOC681820*	7:69915236C>T	*C8orf47*	None	None
NEC	*D3ZFH8_RAT*	7:69922079T>G	*HMGB1P5*	n = 3	n = 3
NEC	*D3ZFH8_RAT*	7:69922335T>C	*HMGB1P5*	None	n = 1
NEC	*Pop1*	7:69974814C>G	*POP1*	None	n = 1
NEC	*RGD1563045*	7:75873231C>T	*SMOC1*	None	None
NEC	*E9PSK6_RAT*	7:80373722A>G	*PKHD1L1*	n = 1	n = 4
NEC	*D3ZPQ1_RAT*	7:113438014A>T	*C8orf31*	n = 2	n = 3
NEC	*Bik*	7:121380074A>G	*BIK*	n = 3	n = 4
NEC	*Bik*	7:121380101A>G	*BIK*	n = 3	n = 4

## Discussion

The genetic architecture underlying some of the most common epilepsies remains undescribed. This study used WGS to identify genetic variants in a validated spontaneous animal model of GGE with absence seizures, the GAERS rat. Like the human condition, the GAERS rat appears to have a complex genetic architecture with the F_2_ progeny of the GAERS and NEC supporting polygenic determinants.

Estimates show that approximately 40% of the euchromatic rat genome is part of the eutherian core (orthologous bases to both mice and humans) [36] with 97.5% of human ion channel genes, the most heavily implicated set of genes in epilepsy, having a characterized rat ortholog [37].

This sequencing study identified genetic variants that were found in the GAERS strain that were absent in the *rattus norvegicus* reference genome (BN rat). This was independently repeated for the NEC strain and here it would be able to detect epilepsy risk factors that might be shared between the GAERS and the BN rat. We subsequently compared the GAERS and NEC strain to find genetic variants that differed between the two inbred lines. Both strains originated from the same outbred Wistar rat colony, which selectively bred based on the presence (GAERS) or absence (NEC) of spontaneously arisen absence seizures and the corresponding SWD signatures.

We then screened strain-specific GAERS and NEC variants with variants found among F_2_ rats with either high- or non-seizing phenotypes [23]. To both identify candidate variants for the seizure phenotype and to remove likely background rat genome variation, we focused on the GAERS-specific and NEC-specific variants that were present in less than ~10% of the previously sequenced open-access rat strains [31, 32]. Subsequently, to better prioritize variants with potential effects on the epilepsy phenotype we screened those candidate variants through the whole genome sequence data generated on four F_2_ rats. We selected two F_2_ rats at each extreme (two high-seizing F_2_ and two non-seizing F_2_) of the GAERS and NEC seizure phenotype spectrum. Through this screen we identified six GAERS-specific variants that were transmitted to the high-seizing but not the non-seizing F_2_ animals. Conversely, we identified fourteen NEC-specific variants that were transmitted to the non-seizing but not the high-seizing F_2_ animals. Beyond the selected for *Cacna1h* missense variant [23] this approach did not identify additional candidate variants of immediate interest to the phenotypes.

In the NEC filtering of the specific variants, three additional common variants (seen in over five existing rat strain sequences–S2 Table) were found in genes that have been associated with epilepsy and segregate with the two non-seizing F_2_ rats; *Abat* (4-aminobutyrate aminotransferase), *Cyp11b3* (11-beta-hydroxylase) and *Cyp11b2* (aldosterone synthase) [3, 12, 38–44]. While we do not yet have data to support any relevance of these observations, the NEC-specific variants are of interest since it is known that many rat strains will develop spontaneous SWDs, particularly during mature adulthood, while inbred NEC do not record any SWDs or absence seizures during the course of their life [13].

Spontaneously occurring animal models of complex human disease present a powerful opportunity to better understand the genetic architecture and disease pathogenesis beyond what is possible from monogenic animal models of disease. This work illustrates the application of whole genome sequencing on an inbred spontaneously-occurring epilepsy rat strain coupled with sequencing high- and non-seizing F_2_ rats to identify a subset of genetic variants that might individually or collectively associate with the epilepsy phenotype. In this study we cannot rule out contributions coming from common variants with small-modest effects on absence epilepsy risk that are shared across many of the currently available rat strains. While we were able to narrow down individual candidate variants based on F_2_ rat sequencing, this study is not equipped to quantitatively assess the contribution of the GAERS and NEC variants to their corresponding phenotypes. Future studies that generate sequence data across the full spectrum of seizure phenotypes observed in the F_2_ rats is required to perform quantitative assessments of possible oligogenic/polygenic interactions.

The approach used to narrow down candidates is applicable to other animal models that have genetic predisposition to either genetic, like WAG/Rij, or acquired epilepsies. For instance, this whole genome sequence approach can be applied to try to identify susceptibility genes to the development of acquired epilepsy in the FAST (kindling-prone) and SLOW (kindling-resistant) rat strains. Like the GAERS and NEC, the FAST and SLOW rats are a result of selective breeding of genetic predisposition to develop focal seizures in the kindling model [45].

## Conclusions

Overall, this study illustrates that the sequencing of naturally occurring inbred animal models of human disease, when a related inbred control strain is available, can facilitate addressing one of the long standing limitations encountered when investigating polygenic diseases in human populations. Additional F_2_ sequencing along with extensive future studies of these SNVs, indels, and structural variants in larger cohorts of carefully characterised GAERS and NEC F_2_ rats could identify additional variants that may contribute to the risk of absence epilepsy, and the contribution that each gene, individually and in combination, has to the variability of seizure characteristics that are observed in the F_2_ rats.

## Supporting information

S1 Table List of genes showing gain or loss of stop codon, or splice acceptor/donor site in GAERS compared to BN reference genome. (XLSX)

S2 Table List of genes showing missense codon specific to NEC compared to BN reference genome (i.e., absent in GAERS). (XLSX)

S3 Table List of genes showing likely-gene-disrupting indels in GAERS genome as compared to BN reference genome. (XLSX)

S4 Table List of genes showing likely-gene-disrupting indels in NEC genome as compared to BN reference genome. (XLSX)

S5 Table Copy number variations (CNVs) between GAERS, NEC and BN genome predicted using ERDS. (XLSX)

S6 Table non-intergenic copy number variants (CNVs) specific to GAERS or NEC genome predicted using ERDS. (XLSX)

## References

[1] Porter RJ. The absence epilepsies. Epilepsia. 1993;34 Suppl 3:S42–8. Epub 1993/01/01. . 8500432 10.1111/j.1528-1167.1993.tb06258.x

[2] Fisher RS, van Emde Boas W, Blume W, Elger C, Genton P, Lee P, et al Epileptic seizures and epilepsy: definitions proposed by the International League Against Epilepsy (ILAE) and the International Bureau for Epilepsy (IBE). Epilepsia. 2005;46(4):470–2. Epub 2005/04/09. doi: 10.1111/j.0013-9580.2005.66104.x . 15816939 10.1111/j.0013-9580.2005.66104.x

[3] Loiseau P, Duche B, Pedespan JM. Absence epilepsies. Epilepsia. 1995;36(12):1182–6. . 7489694 10.1111/j.1528-1157.1995.tb01060.x

[4] Gardiner M. Genetics of idiopathic generalized epilepsies. Epilepsia. 2005;46 Suppl 9:15–20. Epub 2005/11/24. doi: 10.1111/j.1528-1167.2005.00310.x . 16302872 10.1111/j.1528-1167.2005.00310.x

[5] Doose H, Gerken H, Horstmann T, Völzke E. Genetic factors in spike-wave absences. Epilepsia. 1973;14(1):57–75. 4200260 10.1111/j.1528-1157.1973.tb03942.x

[6] Consortium E, Leu C, de Kovel CG, Zara F, Striano P, Pezzella M, et al Genome-wide linkage meta-analysis identifies susceptibility loci at 2q34 and 13q31.3 for genetic generalized epilepsies. Epilepsia. 2012;53(2):308–18. doi: 10.1111/j.1528-1167.2011.03379.x . 22242659 10.1111/j.1528-1167.2011.03379.x

[7] Consortium E, Consortium EM, Steffens M, Leu C, Ruppert AK, Zara F, et al Genome-wide association analysis of genetic generalized epilepsies implicates susceptibility loci at 1q43, 2p16.1, 2q22.3 and 17q21.32. Hum Mol Genet. 2012;21(24):5359–72. doi: 10.1093/hmg/dds373 . 22949513 10.1093/hmg/dds373

[8] Ottman R. Genetic epidemiology of epilepsy. Epidemiol Rev. 1997;19(1):120–8. Epub 1997/01/01. . 9360909 10.1093/oxfordjournals.epirev.a017934

[9] Berkovic SF, Mulley JC, Scheffer IE, Petrou S. Human epilepsies: interaction of genetic and acquired factors. Trends Neurosci. 2006;29(7):391–7. doi: 10.1016/j.tins.2006.05.009 . 16769131 10.1016/j.tins.2006.05.009

[10] Doose H. Absence epilepsy of early childhood—genetic aspects. Eur J Pediatr. 1994;153(5):372–7. Epub 1994/05/01. . 8033930 10.1007/BF01956423

[11] Marescaux C, Vergnes M, Micheletti G, Depaulis A, Reis J, Rumbach L, et al [A genetic form of petit mal absence in Wistar rats]. Rev Neurol (Paris). 1984;140(1):63–6. Epub 1984/01/01. . 6420867

[12] Marescaux C, Vergnes M, Depaulis A. Genetic absence epilepsy in rats from Strasbourg—a review. Journal of neural transmission Supplementum. 1992;35:37–69. Epub 1992/01/01. . 1512594 10.1007/978-3-7091-9206-1_4

[13] Danober L, Deransart C, Depaulis A, Vergnes M, Marescaux C. Pathophysiological mechanisms of genetic absence epilepsy in the rat. Progress in neurobiology. 1998;55(1):27–57. Epub 1998/05/29. . 9602499 10.1016/s0301-0082(97)00091-9

[14] Micheletti G, Warter JM, Marescaux C, Depaulis A, Tranchant C, Rumbach L, et al Effects of drugs affecting noradrenergic neurotransmission in rats with spontaneous petit mal-like seizures. Eur J Pharmacol. 1987;135(3):397–402. Epub 1987/03/31. . 3034636 10.1016/0014-2999(87)90690-x

[15] Liu L, Zheng T, Morris MJ, Wallengren C, Clarke AL, Reid CA, et al The mechanism of carbamazepine aggravation of absence seizures. J Pharmacol Exp Ther. 2006;319(2):790–8. Epub 2006/08/10. doi: 10.1124/jpet.106.104968 . 16895979 10.1124/jpet.106.104968

[16] Zheng T, Clarke AL, Morris MJ, Reid CA, Petrou S, O'Brien TJ. Oxcarbazepine, not its active metabolite, potentiates GABAA activation and aggravates absence seizures. Epilepsia. 2009;50(1):83–7. Epub 2008/08/23. doi: 10.1111/j.1528-1167.2008.01759.x . 18717705 10.1111/j.1528-1167.2008.01759.x

[17] Depaulis A, David O, Charpier S. The genetic absence epilepsy rat from Strasbourg as a model to decipher the neuronal and network mechanisms of generalized idiopathic epilepsies. Journal of neuroscience methods. 2016;260:159–74. doi: 10.1016/j.jneumeth.2015.05.022 26068173 10.1016/j.jneumeth.2015.05.022

[18] Dezsi G, Ozturk E, Stanic D, Powell KL, Blumenfeld H, O'Brien TJ, et al Ethosuximide reduces epileptogenesis and behavioral comorbidity in the GAERS model of genetic generalized epilepsy. Epilepsia. 2013;54(4):635–43. Epub 2013/03/08. doi: 10.1111/epi.12118 ; 23464801 10.1111/epi.12118 PMC3618492

[19] Jones NC, Salzberg MR, Kumar G, Couper A, Morris MJ, O'Brien TJ. Elevated anxiety and depressive-like behavior in a rat model of genetic generalized epilepsy suggesting common causation. Exp Neurol. 2008;209(1):254–60. Epub 2007/11/21. doi: 10.1016/j.expneurol.2007.09.026 . 18022621 10.1016/j.expneurol.2007.09.026

[20] Ott D, Caplan R, Guthrie D, Siddarth P, Komo S, Shields WD, et al Measures of psychopathology in children with complex partial seizures and primary generalized epilepsy with absence. J Am Acad Child Adolesc Psychiatry. 2001;40(8):907–14. doi: 10.1097/00004583-200108000-00012 . 11501690 10.1097/00004583-200108000-00012

[21] Ott D, Siddarth P, Gurbani S, Koh S, Tournay A, Shields WD, et al Behavioral disorders in pediatric epilepsy: unmet psychiatric need. Epilepsia. 2003;44(4):591–7. . 12681010 10.1046/j.1528-1157.2003.25002.x

[22] Rudolf G, Bihoreau MT, Godfrey RF, Wilder SP, Cox RD, Lathrop M, et al Polygenic control of idiopathic generalized epilepsy phenotypes in the genetic absence rats from Strasbourg (GAERS). Epilepsia. 2004;45(4):301–8. Epub 2004/03/20. doi: 10.1111/j.0013-9580.2004.50303.x . 15030491 10.1111/j.0013-9580.2004.50303.x

[23] Powell KL, Cain SM, Ng C, Sirdesai S, David LS, Kyi M, et al A Cav3.2 T-type calcium channel point mutation has splice-variant-specific effects on function and segregates with seizure expression in a polygenic rat model of absence epilepsy. J Neurosci. 2009;29(2):371–80. Epub 2009/01/16. doi: 10.1523/JNEUROSCI.5295-08.2009 . 19144837 10.1523/JNEUROSCI.5295-08.2009 PMC6664949

[24] Powell KL, Tang H, Ng C, Guillemain I, Dieuset G, Dezsi G, et al Seizure expression, behavior, and brain morphology differences in colonies of Genetic Absence Epilepsy Rats from Strasbourg. Epilepsia. 2014 doi: 10.1111/epi.12840 . 25377760 10.1111/epi.12840

[25] Saar K, Beck A, Bihoreau M-T, Birney E, Brocklebank D, Chen Y, et al SNP and haplotype mapping for genetic analysis in the rat. Nature genetics. 2008;40(5):560–6. doi: 10.1038/ng.124 18443594 10.1038/ng.124 PMC5915293

[26] Li H, Durbin R. Fast and accurate short read alignment with Burrows-Wheeler transform. Bioinformatics. 2009;25(14):1754–60. Epub 2009/05/20. doi: 10.1093/bioinformatics/btp324 ; 19451168 10.1093/bioinformatics/btp324 PMC2705234

[27] Li H, Handsaker B, Wysoker A, Fennell T, Ruan J, Homer N, et al The Sequence Alignment/Map format and SAMtools. Bioinformatics. 2009;25(16):2078–9. doi: 10.1093/bioinformatics/btp352 19505943 10.1093/bioinformatics/btp352 PMC2723002

[28] Consortium S, Saar K, Beck A, Bihoreau MT, Birney E, Brocklebank D, et al SNP and haplotype mapping for genetic analysis in the rat. Nature genetics. 2008;40(5):560–6. doi: 10.1038/ng.124 . 18443594 10.1038/ng.124 PMC5915293

[29] Sherry ST, Ward MH, Kholodov M, Baker J, Phan L, Smigielski EM, et al dbSNP: the NCBI database of genetic variation. Nucleic Acids Res. 2001;29(1):308–11. Epub 2000/01/11. ; 11125122 10.1093/nar/29.1.308 PMC29783

[30] Atanur SS, Birol I, Guryev V, Hirst M, Hummel O, Morrissey C, et al The genome sequence of the spontaneously hypertensive rat: Analysis and functional significance. Genome Res. 2010;20(6):791–803. doi: 10.1101/gr.103499.109 ; 20430781 10.1101/gr.103499.109 PMC2877576

[31] Atanur S, Diaz A, Maratou K, Sarkis A, Rotival M, Game L, et al Genome sequencing reveals loci under artificial selection that underlie disease phenotypes in the laboratory rat. Cell. 2013;154(3):691–703. doi: 10.1016/j.cell.2013.06.040 23890820 10.1016/j.cell.2013.06.040 PMC3732391

[32] Hermsen R, de Ligt J, Spee W, Blokzijl F, Schäfer S, Adami E, et al Genomic landscape of rat strain and substrain variation. BMC genomics. 2015;16:357 doi: 10.1186/s12864-015-1594-1 25943489 10.1186/s12864-015-1594-1 PMC4422378

[33] Hinrichs AS, Karolchik D, Baertsch R, Barber GP, Bejerano G, Clawson H, et al The UCSC Genome Browser Database: update 2006. Nucleic Acids Res. 2006;34(Database issue):D590–8. doi: 10.1093/nar/gkj144 ; 16381938 10.1093/nar/gkj144 PMC1347506

[34] Zhu M, Need AC, Han Y, Ge D, Maia JM, Zhu Q, et al Using ERDS to infer copy-number variants in high-coverage genomes. Am J Hum Genet. 2012;91(3):408–21. doi: 10.1016/j.ajhg.2012.07.004 ; 22939633 10.1016/j.ajhg.2012.07.004 PMC3511991

[35] Lemke JR, Riesch E, Scheurenbrand T, Schubach M, Wilhelm C, Steiner I, et al Targeted next generation sequencing as a diagnostic tool in epileptic disorders. Epilepsia. 2012;53(8):1387–98. doi: 10.1111/j.1528-1167.2012.03516.x . 22612257 10.1111/j.1528-1167.2012.03516.x

[36] Gibbs RA, Weinstock GM, Metzker ML, Muzny DM, Sodergren EJ, Scherer S, et al Genome sequence of the Brown Norway rat yields insights into mammalian evolution. Nature. 2004;428(6982):493–521. Epub 2004/04/02. doi: 10.1038/nature02426 . 15057822 10.1038/nature02426

[37] Dwinell MR, Worthey EA, Shimoyama M, Bakir-Gungor B, DePons J, Laulederkind S, et al The Rat Genome Database 2009: variation, ontologies and pathways. Nucleic Acids Res. 2009;37(Database issue):D744–9. doi: 10.1093/nar/gkn842 ; 18996890 10.1093/nar/gkn842 PMC2686558

[38] Kaminski RM, Rogawski MA. 11beta-Hydroxylase inhibitors protect against seizures in mice by increasing endogenous neurosteroid synthesis. Neuropharmacology. 2011;61(1–2):133–7. doi: 10.1016/j.neuropharm.2011.03.019 ; 21458468 10.1016/j.neuropharm.2011.03.019 PMC3105122

[39] Koe AS, Salzberg MR, Morris MJ, O'Brien TJ, Jones NC. Early life maternal separation stress augmentation of limbic epileptogenesis: the role of corticosterone and HPA axis programming. Psychoneuroendocrinology. 2014;42:124–33. doi: 10.1016/j.psyneuen.2014.01.009 . 24636509 10.1016/j.psyneuen.2014.01.009

[40] Maggio N, Segal M. Stress and corticosteroid modulation of seizures and synaptic inhibition in the hippocampus. Experimental neurology. 2012;234(1):200–7. doi: 10.1016/j.expneurol.2011.12.035 22227061 10.1016/j.expneurol.2011.12.035

[41] Edwards H, Vimal S, Burnham WM. The effects of ACTH and adrenocorticosteroids on seizure susceptibility in 15-day-old male rats. Experimental neurology. 2002;175(1):182–90. 12009770 10.1006/exnr.2002.7874

[42] Gol'tvanitsa GA, Shirshov I, Marueva NA, Kritskaia I, Leont'eva EV, Temnikova IV. [Balance of steroid hormones among children and teenagers with epilepsies]. Žurnal nevrologii i psihiatrii imeni SS Korsakova. 2012;112(2):25–8. 22677661

[43] Arion D, Sabatini M, Unger T, Pastor J, Alonso Nanclares L, Ballesteros Yáñez I, et al Correlation of transcriptome profile with electrical activity in temporal lobe epilepsy. Neurobiology of disease. 2006;22(2):374–87. 16480884 10.1016/j.nbd.2005.12.012

[44] Micheletti G, Vergnes M, Marescaux C, Reis J, Depaulis A, Rumbach L, et al Antiepileptic drug evaluation in a new animal model: spontaneous petit mal epilepsy in the rat. Arzneimittel-Forschung. 1985;35(2):483–5. Epub 1985/01/01. . 3922381

[45] Racine RJ, Steingart M, McIntyre DC. Development of kindling-prone and kindling-resistant rats: selective breeding and electrophysiological studies. Epilepsy research. 1999;35(3):183–95. 10413314 10.1016/s0920-1211(99)00013-3

